# Development and validation of a method for analyzing the sialylated glycopeptides of recombinant erythropoietin in urine using LC–HRMS

**DOI:** 10.1038/s41598-023-31030-y

**Published:** 2023-03-08

**Authors:** Yoondam Seo, Jisoo Park, Hyeon-Jeong Lee, Minyoung Kim, Inseon Kang, Junghyun Son, Min-Kyu Oh, Hophil Min

**Affiliations:** 1 Doping Control Center, Korea Institute of Science and Technology, Hwarang-ro 14-gil, Seongbuk-gu, Seoul, 02792 Republic of Korea; 2 Department of Chemical and Biological Engineering, Korea University, Anam-ro, Seongbuk-gu, Seoul, 02841 Republic of Korea; 3 Graduate School of Pharmaceutical Sciences, Ewha Womans University, Seoul, Republic of Korea

**Keywords:** Bioanalytical chemistry, Mass spectrometry

## Abstract

Erythropoietin (EPO) is a glycoprotein hormone that stimulates red blood cell production. It is produced naturally in the body and is used to treat patients with anemia. Recombinant EPO (rEPO) is used illicitly in sports to improve performance by increasing the blood’s capacity to carry oxygen. The World Anti-Doping Agency has therefore prohibited the use of rEPO. In this study, we developed a bottom-up mass spectrometric method for profiling the site-specific N-glycosylation of rEPO. We revealed that intact glycopeptides have a site-specific tetra-sialic glycan structure. Using this structure as an exogenous marker, we developed a method for use in doping studies. The profiling of rEPO N-glycopeptides revealed the presence of tri- and tetra-sialylated N-glycopeptides. By selecting a peptide with a tetra-sialic acid structure as the target, its limit of detection (LOD) was estimated to be < 500 pg/mL. Furthermore, we confirmed the detection of the target rEPO glycopeptide using three other rEPO products. We additionally validated the linearity, carryover, selectivity, matrix effect, LOD, and intraday precision of this method. To the best of our knowledge, this is the first report of a doping analysis using liquid chromatography/mass spectrometry-based detection of the rEPO glycopeptide with a tetra-sialic acid structure in human urine samples.

## Introduction

Erythropoietin (EPO) is a representative glycoprotein hormone. It plays a crucial role in the survival, proliferation, and maturation of erythroid cells and is naturally produced by renal peritubular cells to stimulate red blood cell production^1^. EPO contains approximately 40% carbohydrates on average as well as one O-glycosylation site (Ser^126^) and three N-glycosylation sites (Asn^24^, Asn^38^, and Asn^83^)^2,3^. N-glycosylation is crucial for the biological activity of EPO in vivo as it influences the biosynthesis, secretion, receptor affinity, plasma half-life, and clearance of this hormone. In particular, the terminal sialylation of glycoproteins affects their circulatory half-life in blood^4^. Differences in glycosylation also affect proteins’ biological attributes, including their stability, solubility, antigenicity, folding, and half-lives^5^. Therefore, enhancing the pharmacological properties of EPO, such as an extended plasma half-life, has been a focus of new recombinant EPO (rEPO) development. Two commercially available approaches, namely hyperglycosylation and PEGylation, have been used successfully^6^. Darbepoetin alfa is a hyperglycosylated product that contains five N-linked carbohydrate chains (two more chains than endogenous EPO)^3^. Because of the increased carbohydrate content of darbepoetin alfa, which contains sialic acid, the novel erythropoiesis-stimulating protein differs biochemically from endogenous EPO in that it has an approximately threefold longer serum half-life; thus, it can achieve a comparable biological response with less frequent administration^7^. Epoetin beta is PEGylated with a methoxy polyethylene glycol (PEG) group in continuous erythropoiesis receptor activator. This 30-kDa PEG polymer is chemically bound to an N-terminal amino acid^8^. Because of PEGylation, the receptor affinity decreases, half-life increases by sevenfold, and in vivo activity is higher than that of endogenous EPO^9^.

In terms of sports performance, EPO has been demonstrated to enhance the proliferation of red blood cells and consequently increase the amount of oxygen delivered to muscles. The increased level of oxygen in circulation slows the progression of muscle fatigue, thereby increasing endurance during athletic activities^10^. Therefore, since the availability of rEPO in Europe in 1987, it has been used illicitly in endurance sports. In 1990, this drug was prohibited by the International Olympic Committee Medical Commission. As rEPO became widely available in the 1980s, several techniques have been employed to detect this hormone in urine and determine its illegal use^11,12^. The World Anti-Doping Agency (WADA) has recommended isoelectric focusing and/or sarcosyl-polyacrylamide gel electrophoresis (SAR-PAGE) following the enrichment and purification of EPO to detect illegally used rEPO in urine^13,14^. Because endogenous EPO and rEPO differ in their molecular weights and charges, these methods allow the separation of these compounds according to these differences. Regarding the separation of endogenous EPO and rEPO forms, such as EPO alpha or beta, endogenous EPO contains only mono-, di-, and tri-oligosaccharides and lacks the tetra-sialic acid structure present on the glycans of rEPO^2^. This is the only difference between endogenous EPO against EPO alpha and beta. Consequently, the detection remains ambiguous.

Liquid chromatography–high resolution mass spectrometry (LC–HRMS) has been employed for the detection of rEPO over recent years^15,16^. To analyze protein glycosylation, both glycan- and glycopeptide-based analyses can be used. The former approach involves the detachment of glycans from proteins and their characterization^17^. The glycans are cleaved from glycoproteins enzymatically or chemically and are then purified before MS analysis^18^. Therefore, this approach may not provide glycan information for each glycosylation site if the glycoprotein contains two or more of these sites. In contrast, in glycopeptide-based analysis, the glycans remain attached to the protein sites, enabling the simultaneous elucidation of glycopeptide amino acid sequences, glycosylation site-specific information^19^, and glycan composition^20,21^. In general, glycopeptides are analyzed using a derivatization strategy^22^. However, the derivatization of glycopeptides remains challenging, mostly because of the difficulty of ensuring the uniformity of reactions on the peptide moiety. Therefore, glycopeptide studies are usually conducted with large amounts of protein. However, studies investigating low concentrations of glycopeptides are limited.

In this study, we developed a method for profiling rEPO-specific tetra-sialylated glycopeptides and detecting target glycopeptides at low concentrations, particularly 500 pg/mL (range, 0–800 ng/mL), in urine samples. To profile the intact site-specific sialylated rEPO glycopeptide, we used a method for enriching the sialic acid-containing peptides that takes advantage of the extremely high affinity of titanium dioxide (TiO_2_) toward the sialic acid residues. In addition, to analyze low concentrations of tetra-sialylated glycopeptides in human urine, we used an EPO antibody plate without derivatization or labeling. The development method was validated for linearity, limit of detection (LOD), carryover, selectivity, matrix effects, and intraday precision. Using the developed profiling method, we successfully profiled the site-specific glycosylation of intact glycopeptides and selected a sialylated glycopeptide as an exogenous marker for detecting rEPO. This method enables the identification of intact glycopeptides with site-specific tetra-sialic glycan structures.

## Materials and methods

### Samples

In this study, pooled urine samples were used. We received anonymous urine samples from athletes that were collected by the Korea Anti-Doping Agency for antidoping tests. We only used samples from athletes who had agreed that their samples could be used for research purposes. In total, 150 athletes from different sports disciplines who participated in sports events, tested negative for doping substances at antidoping laboratories in Korea, and provided consent were included in this study. The study was confirmed to be exempt from institutional review board review. The European Directorate for the Quality of Medicines provided EPO Biological Reference Preparation (BRP) batch 5. Epokine Prefilled Injection (10,000 IU/mL; 1 mL) and Aropotin Injection (4000 IU; 0.5 mL) were purchased from HK inno.N (Gyenggi-do, Korea). Espogen Prefilled Injection (1000 IU/mL; 1 mL) was obtained from LG Chem (Gyenggi-do, Korea).

### Reagents and chemicals

An EPO antibody plate was obtained from Stem Cell Technologies (Vancouver, Canada). TiO_2_ beads (Titansphere, particle size: 10 μm) were provided by GL Sciences (Tokyo, Japan). Triethylammonium bicarbonate (TEAB), Tris-(2-carboxyethyl) phosphine (TCEP), ammonium hydroxide (NH_4_OH), trifluoroacetic acid (TFA), and 2-chloroacetamide (CAA) were purchased from Sigma-Aldrich (St. Louis, MO, USA). Glu-C and trypsin were obtained from Promega (Madison, WI, USA). Acetonitrile (ACN) for LC/MS-grade and methanol (MeOH) were provided by AVANTOR (Radnor, PA, USA). Formic acid (FA) was provided by Wako Pure Chemicals (Osaka, Japan). HPLC-grade water, 0.1% FA in ACN, and 0.1% FA in water were purchased from Thermo Fisher Scientific (Waltham, MA, USA). Tris was obtained from Roche (Basel, Switzerland). Further, 15-mL and 0.5-mL 30 k Amicon filters were purchased from Millipore (Billerica, MA, USA). An SDB-RPS disk was obtained from Affinisep (Petit-Couronne, France). Hydrochloric acid (6 N) was purchased from Samchun Pure Chemical (Gyenggi-do, Korea). A C8 disc was purchased from Empore (Oxford, PA, USA).

### Sample preparation for sialylated glycopeptide profiling

To denature the sample, 25 µL of 100 mM TEAB buffer, 5 µL of 100 mM TCEP, and 20 µL of 100 mM CAA were added to the sample, and the solution was heated for 20 min at 95℃. The protein sample was digested for 4 h at 37 °C using Glu-C at a protein:enzyme ratio of 100:1 (w/w). Then, trypsin was added at a protein:enzyme ratio of 100:1, and the solution was incubated at 37 °C for 16 h. The resulting mixture of peptides and glycopeptides was heated at 95 °C for 5 min to stop digestion. For the desalting step, samples were dried using a vacuum centrifuge.

### Sialylated glycopeptide enrichment

To enrich the sialylated intact glycopeptides, TiO_2_ beads were used. First, a TiO_2_ bead slurry was prepared by adding 200 µL of 100% ACN to a 1-mL low-binding tube containing 20 mg of TiO_2_. Then, 60 µL of the slurry was dried using a vacuum centrifuge for 10 min at 25 °C. Overall, 1 mL of TiO_2_ loading buffer (5% TFA, 80% ACN in 1 M glycolic acid) and 6 mg of TiO_2_ were added to 1 mg of the peptide mixture. The mixture was placed on a mixer, incubated for 30 min, and centrifuged for 1 min at 1000 × *g* and room temperature. The supernatant was discarded, and 400 µL of the loading buffer was added to the beads. After placing the beads on the mixer for 1 min, they were centrifuged for 1 min in a micro-centrifuge, and the supernatant was discarded. Next, 400 µL of washing buffer 1 (80% ACN, 1% TFA) was added to the beads, and the mixture was vortexed for 15 s, followed by the addition of 400 µL of washing buffer 2 (20% ACN, 0.1% TFA). The beads were dried for 5–10 min in a vacuum centrifuge. To elute sialylated glycopeptides, the beads were incubated in 200 µL of TiO_2_ elution buffer (25% NH_4_OH) for 20 min on the mixer at room temperature. The TiO_2_ beads were removed using a P200 pipette tip plugged with C8 material. The beads were washed with 10 µL of 50% ACN. The eluate was dried using vacuum centrifuge and stored at − 80℃ until instrument analysis.

### Sialylated glycopeptide profiling of rEPO using LC

The DIONEX Ultimate 3000 RSLC nano System for LC was provided by Thermo Fisher Scientific. Chromatographic separation was performed using RSLC C18 (75 µm × 50 cm), RSLC C18 (75 µm × 15 cm), and Acclaim PepMap (75 µm × 2 cm) columns. We used 0.1% FA and 2% ACN in water as mobile phase A and 0.1% FA in 80% ACN as mobile phase B. The temperature of column oven was set at 50 °C, and the flow rate of the mobile phase was 300 nL/min.

For profiling, gradient elution was performed using the following steps: 5 min of isocratic flow in 2% mobile phase B; 5% mobile phase B linear to 35% mobile phase B until 85 min; 35% mobile phase B linear to 90% mobile phase B until 95 min; 5 min of isocratic flow in 95% mobile phase B; 90% mobile phase B linear to 2% mobile phase B until 102 min; and 18 min of isocratic flow in 2% mobile phase B (total run time, 120 min).

### Sialylated glycopeptide profiling of rEPO using MS

For profiling, the samples were analyzed via Q-Exactive plus mass spectrometer (Thermo Fisher Scientific). The capillary temperature was set at 275 °C. The ion spray energy was set at 2.0 kV in the positive mode. The auxiliary, sheath, and sweep gas flow rates were set to 0 arbitrary units. For the full MS mode, the mass scan was analyzed at a resolution of 70,000. The automatic gain control (AGC) value was set to 1 × 10^6^, and the maximum injection time was 50 ms. The scan range was 350–1600 m/z. For the full ms/ddms^2^ mode, the MS/MS scan was analyzed at a resolution of 35,000. The maximum injection time was set to 80 ms, and the AGC value was set to 1 × 10^6^. The isolation window for MS/MS was 1.6 m/z.

### Data analysis for glycopeptide identification

For glycopeptide profiling, Byonic software (v 3.10.2. Protein Metrics)^23^ and pGlyco version 2.2.2^24^ software were employed. In Byonic software, the fragmentation type was set to QTOF/HCD, and the precursor mass tolerance was set to 10 ppm. The cleavage site was EDRK, cleavage side was the C-terminus, and fragment mass tolerance was set to 10 ppm. The digestion specificity was set to the fully specific (fastest) mode. Missed cleavages were set to 2. The protein false discovery rate (FDR) was 1% or 20 reverse count. The maximum precursor mass was 10,000, and the maximum number of precursors per MS/MS was 5. The threshold score was set to 300 and the glycan list was set to N-glycan 47 rEPO provided by Byonic^25^.

The results of pGlyco software were compared with those of Bionic software. The fragmentation type was set to HCD, and the precursor mass tolerance was set to 10 ppm. The cleavage site was EDRK, cleavage side was the C-terminus, and fragment mass tolerance was set to 10 ppm. Missed cleavages were set to 2. The protein FDR was 1% or 20 reverse count. The threshold score was set to 10 and the maximum precursor mass was 10,000, and the maximum number of precursors per MS/MS was 5. The glycan list was set to the category of large mouse provided by pFind studio^26^.

### Immunoaffinity purification of EPO from urine

For immunoaffinity purification, 15 mL of urine samples and 1.5 mL of 3.75 M Tris–HCl (pH 7.4) were added to a 50-mL conical tube. The mixture was centrifuged for 20 min at 2,800 × *g* and 4 °C. The urine samples were transferred to Amicon Ultra 15-mL filters and centrifuged for 20 min at 4 °C and 2800 × *g*. The filtered sample (< 30 kDa) was discarded. Next, 15 mL of 50 mM Tris–HCl (pH 7.4) was added to the samples, and the mixture was centrifuged twice for 20 min at 2800 × *g* and 4 °C. The samples were then transferred to 0.5-mL Amicon Ultra filters and centrifuged for 10 min at 14,000 × *g* and 4 °C. Next, the samples were transferred to new collection tubes and centrifuged for 2 min at 2000 × *g*. All retentates were transferred to the StemCell EPO antibody plate, covered with an adhesive film, and incubated at 37 °C for 1 h. The samples were washed five times with 300 µL of 100 mM TEAB. They were dried until the solvent on the plate had completely evaporated. Subsequently, 50 µL of the sample eluting buffer (100 mM TEAB, 10 mM TCEP, and 40 mM CAA) was added to each well of the EPO antibody plate. Elution, denaturation, reduction, and alkylation were performed at 95 °C in a thermomixer for 20 min. The samples were centrifuged at 2800 × *g* for 2 min and 4 °C. Enzyme digestion was performed according to the digestion method described in “Sample preparation for sialylated glycopeptide profiling” section.

### Detection of rEPO sialylated glycopeptide using LC

In the target-selected ion monitoring (t-SIM)/ddms^2^ mode for detecting the target glycopeptide, the gradient elution process consisted of the following steps: 12% mobile phase B linear to 22% mobile phase B until 28.5 min; 35% mobile phase B linear to 95% mobile phase B until 29 min; 2 min of isocratic flow in 95% mobile phase B; 95% mobile phase B linear to 2% mobile phase B until 31.1 min; and 4 min of isocratic flow in 2% mobile phase B (total run time, 35 min). After cooling the samples, enzyme digestion was performed as previously described.

### Detection of rEPO sialylated glycopeptide using MS

The samples were analyzed for the presence of the target glycopeptide using Exploris 240 mass spectrometer (Thermo Fisher Scientific). The capillary temperature was set at 275 °C. The voltage of ion spray was 1.8 kV in the positive mode. The auxiliary, sheath, and sweep gas flow rates were set to 0 arbitrary units. For the t-SIM mode, the mass scan was analyzed at a resolution of 120,000. The AGC value was set to 2 × 10^6^, and the maximum injection time was 200 ms. In the MS/MS mode, the isolation window was 2 m/z and HCD Stepped collision energies were 10, 20, and 30 V. The resolution was set to 120,000, AGC value was set to 2 × 10^5^, and maximum injection time was 200 ms.

### Method validation

The LOD, carryover, selectivity, matrix effect, linearity, and intraday precision were included in the validation results. The minimum detectable amount was defined as the LOD. The signal-to-noise (S/N) ratio was the peak intensity relative to the background noise. The threshold of the S/N ratio was > 3, and the reference value was > 5. For selectivity, five samples were prepared at a concentration of 10 ng/mL, and five samples from different athletes were used as negative urine samples. The spiked and negative urine samples were analyzed alternately. To determine the impact of the matrix effect, the peak area of a fortified urine was compared with that of a fortified neat sample solution (100 mM TEAB). For linearity evaluation, samples at concentrations of 0, 25, 50, 100, 200, 400, 600, and 800 ng/mL were used. Seven replicates were prepared for each concentration. For carryover measurements, five samples were prepared at a concentration of 1000 ng/mL. The analysis was performed for the urine and blank (MeOH) samples alternately. Five replicates of each urine sample concentration (0.5, 50, and 200 ng/mL) were examined to assess the intraday precision. The results were expressed as %CV of the calculated concentrations. For intraday reproducibility, %CV should be < 15%.

## Results and discussion

### Experimental workflow for sialylated glycopeptide profiling and exogenous sialylated glycopeptide analysis

**Figure 1:**
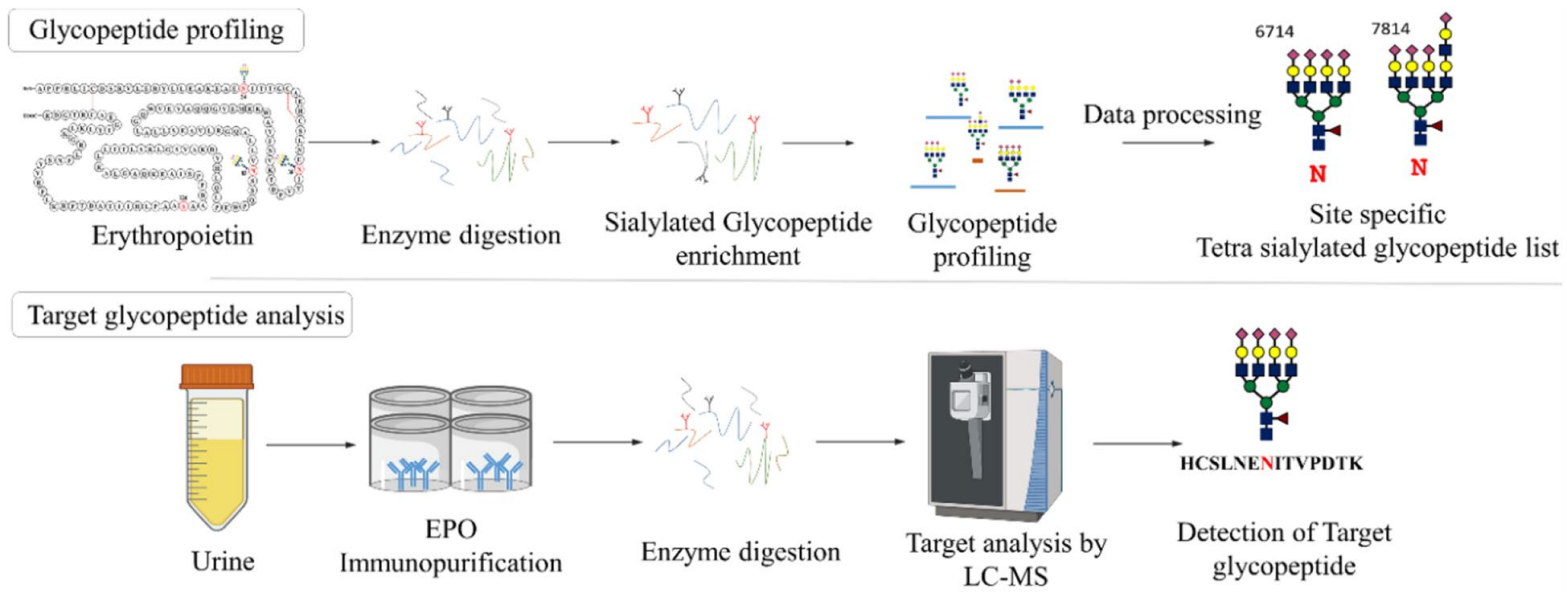
Experimental workflow for glycopeptide profiling and the target analysis method. Profiling for sialylated glycopeptides using TiO_2_ enrichment and target analysis with immunopurification were performed.

EPO, a representative N-glycoprotein, is a 30.4-kDa glycoprotein hormone composed of 165 amino acid residues and three N-glycans^1^. The N-linked oligosaccharides and their branching patterns affect the pharmacodynamics, speed of catabolism, and biological activity of EPO^27,28^. We used BRP from the European Pharmacopoeia as an rEPO to determine the potency of EPO preparations in vivo^29^. BRP contains epoetin alpha and epoetin beta in a 50:50 mixture. It is structurally identical to endogenous EPO and does not differ in its amino acid sequence at the N–O glycosylation site^30,31^. Furthermore, other types of rEPO, such as AROPOTIN®, ESPOGEN®, and EPOKINE®, do not differ from endogenous EPO in terms of the amino acid sequence. To identify the target glycopeptide, we first profiled rEPO and then selected a glycopeptide based on the results. The workflow of LC/MS analysis of the N-glycosylation of rEPO is presented in Fig. 1.

### Profiling of site-specific sialylated rEPO glycopeptides for exogenous glycopeptide analysis

An optimal digestion strategy is required for site-specific profiling. There is no cleavage site between Asn^24^ and Asn^38^; if glycans are present in this region, the peptide produced via trypsin digestion is extremely large for MS analysis. Therefore, we separated the two glycosylation sites using Glu-C. However, Glu-C digestion at the Asn^83^ site produced a large peptide. The glycopeptide obtained following digestion by each of the three enzymes is presented in Table S1. Therefore, we performed multienzyme digestion of rEPO with both Glu-C and trypsin. Through this approach, rEPO glycopeptides with suitable molecular weights and charges were generated for MS detection. Table 1 presents the results of glycopeptide profiling using a three-enzyme digestion strategy.

**Table 1:** Results of glycan profiling through a comparison of three enzymes.

Site	Glycan structure	PMI-Byonic (v3.10.2)			pGlyco (v.2.2.2)		
		Trypsin	Glu-C	Glu-C and trypsin	Trypsin	Glu-C	Glu-C and trypsin
N24	HexNAc(6)Hex(7)Fuc(1)NeuAc(4)					O	O
	HexNAc(7)Hex(8)Fuc(1)NeuAc(4)						O
N38	HexNAc(5)Hex(6)Fuc(1)NeuAc(3)		O	O		O	O
	HexNAc(6)Hex(7)Fuc(1)NeuAc(3)		O	O		O	O
	HexNAc(6)Hex(7)Fuc(1)NeuAc(4)			O		O	O
	HexNAc(7)Hex(8)Fuc(1)NeuAc(3)			O			O
	HexNAc(7)Hex(8)Fuc(1)NeuAc(4)			O		O	O
N83	HexNAc(5)Hex(6)Fuc(1)NeuAc(3)	O		O	O		O
	HexNAc(5)Hex(6)Fuc(1)NeuAc(3) Acetyl(1)	O					
	HexNAc(6)Hex(7)Fuc(1)NeuAc(3)	O			O		
	HexNAc(6)Hex(7)Fuc(1)NeuAc(4)	O	O	O	O		O
	HexNAc(6)Hex(7)Fuc(1)NeuAc(4) Acetyl(1)	O					
	HexNAc(6)Hex(7)Fuc(1)NeuAc(4) Acetyl(2)	O					
	HexNAc(7)Hex(8)Fuc(1)NeuAc(3)	O		O	O		O
	HexNAc(7)Hex(8)Fuc(1)NeuAc(4)	O	O	O	O		O
	HexNAc(8)Hex(9)Fuc(1)NeuAc(4)			O			

HexNAc, N-acetylhexosamine; Hex, Hexose; Fuc, Fucose; NeuAc, Sialic acid.

As discussed in the “Materials and methods” section, we used Byonic software and pGlyco 2.2.2 software to determine the efficiency of the digestion strategy for glycopeptides. We identified the most sialylated glycopeptides via multienzyme digestion with Glu-C and trypsin. In addition, we identified sialylated glycopeptides from specific Asparagine (N) -sites in greater abundance than that obtained via a single-enzyme digestion strategy. After trypsin digestion, peptides containing 8 of 47 glycans at the Asn^83^ site were detected. Following digestion with Glu-C, peptides containing 4 of 47 glycans at the Asn^38^ site and 2 glycans at the Asn^83^ site were detected. Further, through multienzyme digestion with trypsin and Glu-C, a glycopeptide containing 2 of 47 glycans at the Asn^24^ site, 5 glycans at the Asn^38^ site, and 5 glycans at the Asn^83^ site was detected. Sialic acid can bind to TiO_2_ at multiple points, similar to multidentate binding, which may be useful for the selective enrichment of glycopeptides containing sialic acid. TiO_2_ exhibits high affinity toward negatively charged molecules, such as phosphorylated^32^ or sialylated^33^ molecules. Using the profiling method, we selectively enriched and characterized the sialylated glycopeptides. All peaks that exceeded the threshold score were characterized as sialylated glycopeptides using the software, and these peaks contained both tri- and tetra-sialic acids. TiO_2_ enrichment led to the identification of mono-, di-, tri-, and tetra-sialylated glycopeptides, but mono- and di-sialylated glycopeptides could not be identified because they did not reach the threshold (Table S2).

**Figure 2:**
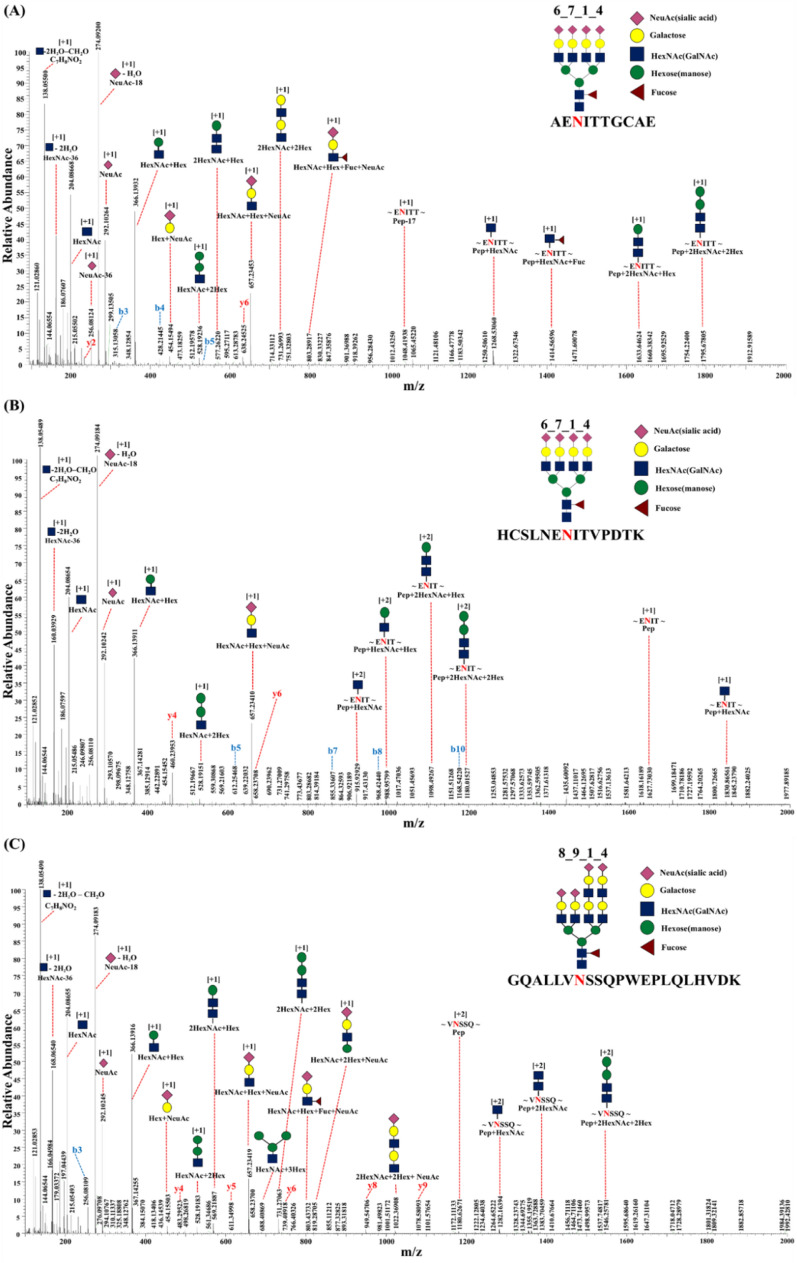
Site-specific MS/MS spectra of a representative N-glycopeptide of recombinant EPO. The nomenclature of the N-glycopeptides is as follows: (**A**) AENITTGCAE_6_7_1_4, (**B**) HCSLNENITVPDTK_6_7_1_4, and (**C**) GQALLVNSSQPWEPLQLHVDK_8_9_1_4. The digits following the peptide sequence represent the number of hexose, N-acetylglucosamine, fucose, and N-acetylneuraminic acid residues, respectively. Red-letter N: N-glycosylation site.

Consequently, the selected target was identified as a tetra-sialylated glycopeptide with a structure that is not observed in endogenous EPO (Table 1)^2^. Furthermore, this tetra-sialylated glycopeptide exhibited a glycan structure, consistent with the results of a previous analysis on EPO glycan structure^34^. Accordingly, we considered the three glycopeptides tetra-sialylated at the Asn^38^ site and three glycopeptides tetra-sialylated at the Asn^83^ site as potential targets. Figure 2 presents the MS/MS spectra of the representative tetra-sialylated glycopeptides. The spectra revealed oxonium ions, glycan-cleaved glycopeptide fragment ions, and peptide-backbone fragment ions, which are the typical fragments of an N-glycopeptide. In particular, the following m/z values were noted in all targets: 204.08 (hexose, + 1), 274.09 (sialic acid-H_2_O, + 1), 292.10 (sialic acid, + 1), 366.139 (N-acetylglucosamine [HexNAc] + hexose, + 1), 657.23 (HexNAc + hexose + sialic acid, + 1), and 658.23 (y6, + 1). These values were used to determine the LOD of BRP and rEPO biosimilars.

### Target glycopeptide analysis of rEPO in urine via immunopurification

We identified site-specific target tetra-sialylated glycopeptide candidates through the above-mentioned rEPO profiling analysis and selected the glycopeptide that appeared to have optimal peak intensity in repeated experiments for use as the rEPO target. The target glycopeptide (HCSLNEN*ITVPDTK_6_7_1_4) was identified with a threshold of 400 points or higher for the Byonic profiling results and 40 points or higher for the pGlyco profiling results. As presented in Table 2, the target peak for rEPO detection represents a glycopeptide containing a tetra-sialic structure at the Asn^38^ site, and the peak for rEPO detection represents the oxonium ion MS/MS peaks of hexose, HexNAc, and sialic acid. We modified the sample preparation and instrumentation methods for target glycopeptide analysis. To detect the rEPO target glycopeptide in urine samples, we first performed immunopurification using an antibody plate. To reduce losses during the experiment, we performed target purification, sample denaturation, reduction, and alkylation on the plate. Second, we modified the instrumentation method by changing the profiling time from 130 to 35 min for target analysis. We analyzed the target glycopeptide using t-SIM to ensure that the t-SIM mode collects data only at the masses of interest rather than collecting data from a wide range of masses. Thus, the t-SIM mode allows the mass spectrometer to detect specific compounds with extremely high sensitivity.

**Table 2:** Transition used for the target analysis.

Site	Target for rEPO detection	Glycan structure	Precursor ion, m/z (charge)	Product ions, m/z (+ 1)
ASN^38^	HSCLNE**N**ITVPDTK		1324.02 (+ 4)	204.08 (Hexose), 274.09 (NeuAc-H_2_O), 292.10 (NeuAc), 366.139 (HexNAc + Hex), 657.23 (HexNAc + Hex + NeuAC), 658.23 (y6)

**Figure 3:**
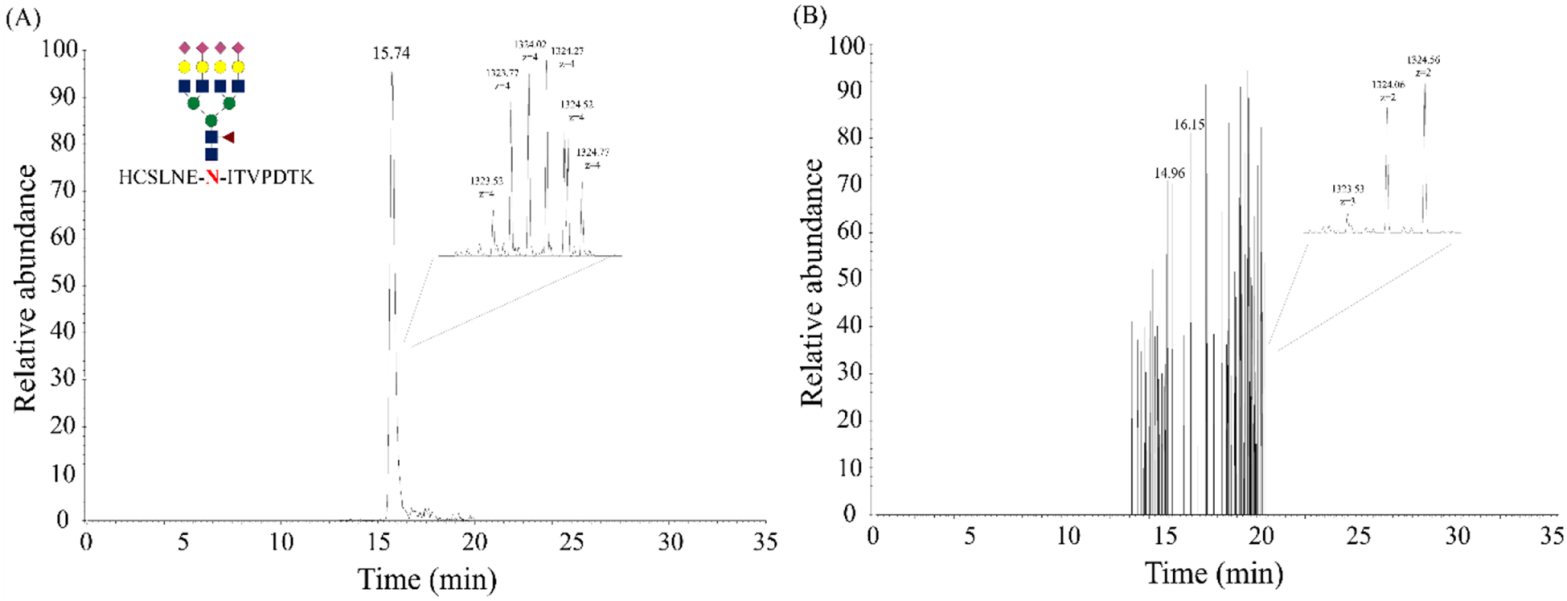
Target glycopeptide analysis using immunopurification. (**A**) Information about the target glycopeptide is provided. Red letters: N-glycosylation site. (**B**) Negative control urine.

The target glycopeptide was not detected in negative control urine (Fig. 3B). Figure 3A presents the results of target glycopeptide analysis. The target could be detected using the 35-min analysis. The presence of the product ions was confirmed by comparing the obtained MS/MS profiling data with those of the corresponding peak.

### Validation of the target glycopeptide analysis method

We performed validation tests to evaluate the repeatability of the developed methods, including immunoprecipitation and Glu-C and trypsin digestion. The validation results included the linearity, selectivity, matrix effect, carryover, LOD, and intraday precision of the methods. A summary of these results is presented in Table S3.

#### Linearity

Eight concentrations were used for the linearity evaluation: 0, 25, 50, 100, 200, 400, 600, and 800 ng/mL. For each concentration, seven replicates were prepared. The linearity reference value (r^2^) was 0.988.

#### Carryover

For the carryover test, five samples were prepared at a concentration of 1,000 ng/mL. The analysis was performed for the urine and blank samples alternately. Carryover was confirmed if the urine sample peak area:blank run peak area ratio was < 5%.

#### Intraday precision

Five replicate urine samples were prepared for each concentration of 0.5, 50, and 200 ng/mL on 3 separate days to evaluate intraday precision. Daily repeatability is indicated by %CV of < 15%. The %CV values for each concentration were as follows: 1.94% for 0.5 ng/mL, 9.48% for 50 ng/mL, and 11.29% for 200 ng/mL.

#### Selectivity

Five urine samples spiked to a concentration of 10 ng/mL and five negative urine samples obtained from different athletes were analyzed alternately. The peak was detected in the blank urine sample.

#### Matrix effect

Five fortified urine samples and five fortified neat sample solutions were analyzed alternately. Matrix effects were not observed in the target peptides of rEPO.

#### LOD

To determine the LOD, we spiked separate samples with different concentrations of rEPO. Efficient purification of the trace amounts of EPO enabled us to attain an LOD of 500 pg/mL for BRP. To measure the LOD, five samples were prepared. These validation results confirmed the effectiveness of the developed target glycopeptide analysis method (Table S3).

### Analysis of rEPO biosimilars

**Figure 4:**
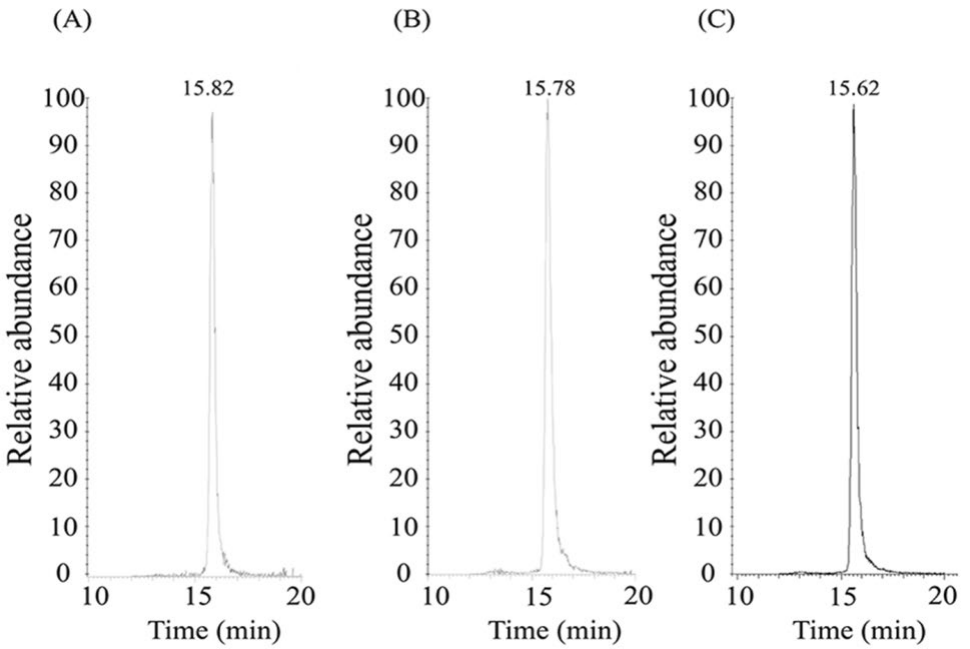
Target glycopeptide analysis for AROPOTIN®, ESPOGEN®, and EPOKINE® at 100 ng/mL. MS spectra of (**A**) AROPOTIN®, (**B**) ESPOGEN®, and (**C**) EPOKINE®. The target glycopeptide is HCSLNENITVPDTK_6_7_1_4.

rEPO biosimilars are the copies of EPO drugs developed after the expiry of the first patent. Although the same amino acid sequence is produced, different production processes of biosimilars can result in minor structural variations from the original drug. Quantitative and qualitative variability might occur in post-translational modifications, such as glycosylation and sialylation. Variation in the N-glycosylation profiles of rEPOs is important because it dramatically affects the drug’s half-life in circulation. Electrophoretic profiles, which are used to detect the presence of rEPO in a doping control sample, may also be affected by structural modification. Therefore, we confirmed the detection of our target rEPO glycopeptide using three rEPO products from other manufacturers. As presented in Fig. 4, we could detect product ions from the glycopeptides targeted for AROPOTIN®, ESPOGEN®, and EPOKINE®. Then, validation of the optimized analysis method was performed. Oxonium and peptide-backbone fragment ions from the target glycopeptide were detected in all three rEPOs (Fig. S1). This result was obtained at a concentration of 100 pg/mL, demonstrating that the method was sensitive even when low sample concentrations were used. Thus, our method for rEPO-specific glycopeptide target analysis yields reliable results.

## Conclusion

In this study, we performed profiling and target analysis of site-specific glycopeptides from rEPO without derivatization both in a standard solution and human urine samples; moreover, we applied this method to analyze three biosimilars. We demonstrated the utility of target analysis of proteins within the detection range of 100–500 pg/mL. We employed a high-sensitivity mass spectrometer to detect the target glycopeptide at low concentrations, based on MS/MS spectra displaying oxonium ions, glycans, glycan-cleaved glycopeptide fragment ions, and peptide-backbone fragment ions.

We aimed to develop a method for doping analysis. We attempted to analyze rEPO without using complicated methods, including derivatization. However, this doping analysis method was limited by the ionization efficiency of the underivatized glycopeptide and the ratio of the target tetra-sialylated glycopeptide to the total rEPO concentration. Therefore, the method has a higher detection limit than the Minimum Requested Performance Level required by the WADA (1 IU/L for rEPO in a urine matrix). This study attempted to develop a method by incorporating target glycopeptides in doping analysis, rather than immediately applied to doping analysis.

Endogenous and exogenous EPOs cannot be distinguished via target peptide analysis using the deglycosylation method. In addition, methods for analyzing glycans derived from deglycosylation cannot completely guarantee that they are derived from EPO when using urine or serum samples. Therefore, this is the first study to perform site-specific detection of low levels of exogenous glycopeptides in urine samples via the immunopurification-based targeted assay.

## Supplementary Information

Supplementary Information.

## Data Availability

Data are available via ProteomeXchange with identifier PXD038932.

## Abbreviations

ACN: Acetonitrile
BRP: Biological reference preparation
CAA: 2-Chloroacetamide
EPO: Erythropoietin
ESI–MS: Electrospray ionization–mass spectrometry
FA: Formic acid
HexNAc: N-acetylhexosamine
LC: Liquid chromatography
MeOH: Methanol
MS: Mass spectrometry
HRMS: High resolution mass spectrometry
ddms^2^: Data-dependent MS/MS
rEPO: Recombinant erythropoietin
t-SIM: Target-selected ion monitoring
TEAB: Triethylammonium bicarbonate
TCEP: Tris-(2-carboxyethyl) phosphine
TFA: Trifluoroacetic acid
