# Supplementary material for: Development and validation of a method for analyzing the sialylated glycopeptides of recombinant erythropoietin in urine using LC–HRMS
Source: Sci Rep. 2023 Mar 8;13:3860. doi: 10.1038/s41598-023-31030-y (PMC9995342; doi:10.1038/s41598-023-31030-y)
Supplement: Supplementary file 1 — Supplementary Information. [file 41598_2023_31030_MOESM1_ESM.docx]

**
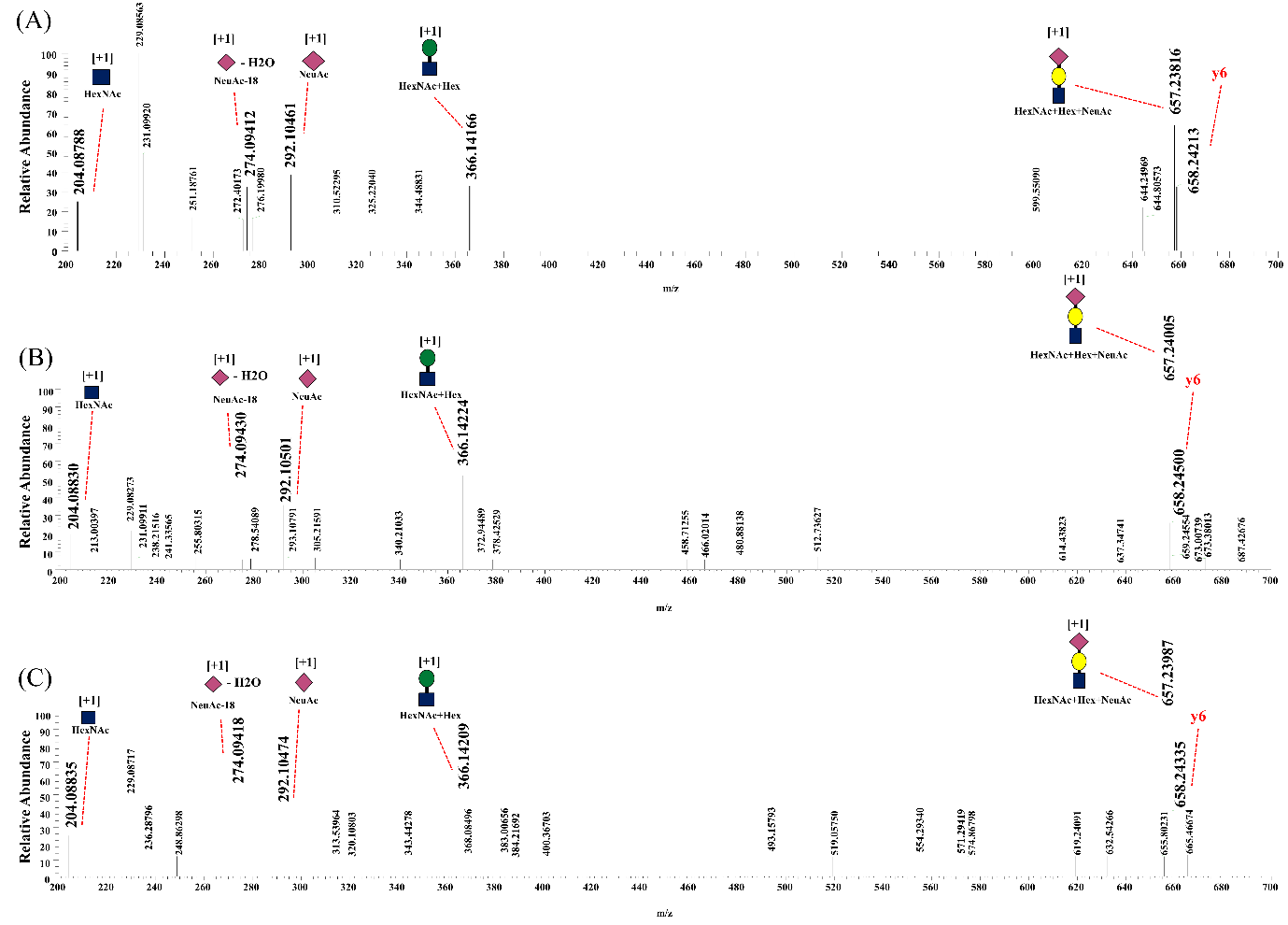
**

**Figure S1.** Oxonium ions and peptide-backbone fragment ions from the target glycopeptide.

MS/MS spectrum of (A) AROPOTIN®, (B) ESPOGEN®, and (C) EPOKINE®, respectively.

Bold letter is transition used for target analysis

**Table S1.** Glycopeptides using 3 types of enzyme in rEPO

| **Enzyme** | **N-glycosylation site** | | |
| --- | --- | --- | --- |
|  | **ASN^24^** | **ASN^38^** | **ASN^83^** |
| Trypsin (K/R) | EAE**N**ITTGCAEHCSLNE**N**ITVPDTK | | GQALLV**N**SSQPWEPLQLHVDK |
| Glu-C (D/E) | AE**N**ITTGCAE | HCSLNE**N**ITVPD | AVLRGQALLV**N**SSQPWEPLQLHVD |
| Trypsin+Glu-C (E/K/R) | AE**N**ITTGCAE | HCSLNE**N**ITVPDTK | GQALLV**N**SSQPWEPLQLHVD |

**Table S2.** The results of glycopeptide profiling using Byonic and pGlyco software

| **Site** | **Peptide** | **Glycan structure** | **Glycan type** | **Observed m/z** | **z** | **Precuror (M+H)** | **Byonic Score** | **Mass error (ppm)** | **pGlyco Score** | **Mass error (ppm)** |
| --- | --- | --- | --- | --- | --- | --- | --- | --- | --- | --- |
|  |  |  |  |  |  |  |  |  |  |  |
| N24 | E NITTGCAE | HexNAc(6)Hex(7)Fuc(1)NeuAc(4) | C[+58], N[+4028] | 1182.9407 | 4 | 4728.741 | N.D | N.D | 46 | 0.8 |
|  | E NITTGCAE | HexNAc(7)Hex(8)Fuc(1)NeuAc(4) | C[+58], N[+3663] | 1274.2245 | 4 | 5093.876 | N.D | N.D | 26 | 1.3 |
| N38 | E.NITVPDTK.V | HexNAc(6)Hex(7)Fuc(1)NeuAc(4) | N[+3663] | 1517.5951 | 3 | 4550.771 | 443 | 0.4 | 33 | 1.1 |
|  | E.NITVPDTK.V | HexNAc(7)Hex(8)Fuc(1)NeuAc(4) | N[+4028] | 1229.7326 | 4 | 4915.909 | 378 | 1.5 | 36 | 1.5 |
|  | E.HCSLNENITVPDTK.V | HexNAc(6)Hex(7)Fuc(1)NeuAc(4) | C[+58], N[+3663] | 1323.7693 | 4 | 5292.055 | 411 | 2.1 | 42 | 1.5 |
| N83 | R.GQALLVNSSQPWEPLQLHVDK.A | HexNAc(6)Hex(7)Fuc(1)NeuAc(4) | N[+3663] | 1506.3897 | 4 | 6022.537 | 456 | 1.8 | 22 | 1 |
|  | R.GQALLVNSSQPWEPLQLHVDK.A | HexNAc(7)Hex(8)Fuc(1)NeuAc(4) | N[+4028] | 1278.1387 | 5 | 6386.664 | 400 | 1.5 | 23 | 0.6 |
|  | R.GQALLV**N**SSQPWEPLQLHVDK.A | HexNAc(8)Hex(9)Fuc(1)NeuAc(4) | N[+4394] | 1351.3650 | 5 | 6752.796 | 424 | 0.8 | N.D | N.D |

N.D: Not detected

**Table S3.** The results of validation

| **Carry over** | Not detected |
| --- | --- |
| **Matrix effect** | Not detected |
| **Linearity** | r^2^=0.9881 |
| **LOD (pg/mL)** | 500 |
| **Intra-day precision (%)** | 0.5 ng/mL (1.94) |
|  | 50 ng/mL (9.48) |
|  | 200 ng/mL (11.29) |
